# Clinical comparison of propofol-remifentanil TCI with sevoflurane induction/maintenance anesthesia in laparoscopic cholecystectomy

**DOI:** 10.12669/pjms.305.5196

**Published:** 2014

**Authors:** Xiaoqian Deng, Tao Zhu

**Affiliations:** 1 Xiaoqian Deng, MD, Resident, Department of Anesthesiology, West China Hospital of Sichuan University, Chengdu, Sichuan, China.; 2 Tao Zhu, MD, Professor, Department of Anesthesiology, West China Hospital of Sichuan University, Chengdu, Sichuan, China.

**Keywords:** TIVA, Propofol-remifentanil, TCI, VIMA, Sevoflurane

## Abstract

***Objective*** **: **We aimed to compare the anesthetic characteristics between total intravenous anesthesia (TIVA) using propofol-remifentanil with target control infusion (TCI) and volatile induction and maintenance anesthesia (VIMA) using sevoflurane and sufentanyl for patients undergoing laparoscopic cholecystectomy.

***Methods: ***A total of 120 patients undergoing laparoscopic cholecystectomy were randomly assigned to two groups. Patients in group T received TCI of propofol-remifentanil for induction and maintenance. Patients in group S received sevoflurane-sufentanyl for induction and maintenance.

***Results:*** Patients in group S had a significantly faster induction time than patients in group T (109s vs*.*44s). The emergence time in terms of time to extubation was comparable between the two groups, while the time to eyes opening (419s vs.483s, p=0.006) and duration in PACU were longer in group S (44 min vs.53 min, p=0.017). Ten (17.2%) patients in group S were administered an antihypertensive drug when gallbladder issues were present, while only 1(1.7%) patient needed this drug in group T (p=0.004).More patients in group T than in group S received fentanyl for analgesia in PACU (88%vs.70%, p=0.013). The incidence of postoperative nausea and vomiting (PONV) in PACU was higher in group S than in group T (20% vs.38%, p=0.027).

***Conclusion: ***Both techniques had advantages and disadvantages in laparoscopic cholecystectomy; none of the techniques studied was superior.

## INTRODUCTION

Anesthesia for ambulatory surgery requires a smooth and pleasant induction, good control of intra-operative conditions, rapid recovery and minimal postoperative side effects. Total intravenous anesthesia (TIVA) with the short-acting anesthetics propofol and remifentanil is characterized by hemodynamic stability and a better recovery profile.^1^ With better control of plasma concentration levels, the introduction of commercially available pumps utilizing target-controlled infusion (TCI) techniques has led to increased safety and predictable timing. The relatively low blood/gas partition coefficient of sevoflurane also provides for both rapid induction and emergence from anesthesia and more rapid control of anesthetic depth, while its non-pungency makes mask induction possible in adults.^2^ Sevoflurane is an excellent induction drug in needle-phobic patients, pediatric patients, and patients with a potentially difficult airway. Its role in these special situations has been extremely helpful.^3^

Both techniques have been shown to be useful for short-duration surgery. There have been several studies comparing propofol with sevoflurane for induction, maintenance, and recovery. However, TIVA using TCI with propofol and remifentanil vs. volatile induction/maintenance anesthesia (VIMA) with sevoflurane and sufentanyl among patients undergoing laparoscopic cholecystectomy has seldom been compared.

We hypothesized that TCI with propofol-remifentanil might be superior to sevoflurane with regard to the speed of induction and recovery, side effects, and hemodynamic stability.

## METHODS

This prospective randomized clinical trial was approved by the ethics committee of Sichuan University in China and was registered on the Chinese Clinical Trial Registry (www.chictr.org/cn, registration number: ChiCTR-TRC-13003834). Written informed consent was obtained from all patients.

The data collection was performed from November to December 2013.A total of 120 non-premedicated patients (ASA physical status I-II, aged<65yr) undergoing laparoscopic cholecystectomy were recruited. Patients with known cardiovascular, respiratory, or metabolic diseases, allergies to any anesthetic agent, impaired renal or hepatic function, morbid obesity, history of alcohol or drug abuse and illiteracy were excluded from participating in this study. Patients were withdrawn from the trial if their safety was threatened at any time.

All participants were randomly assigned to the TIVA group (TCI with propofol and remifentanil) or the VIMA group (sevoflurane and sufentanyl) using a concealed allocation approach (computer-generated codes) utilizing opaque, sealed envelopes containing the randomization schedule. These envelopes were opened immediately before induction of anesthesia.

Personnel who made intraoperative and postoperative observations were not involved in the care-giving process, and personnel in the post-anesthesia care unit (PACU) remained blinded to the randomization. Patients were blinded to the anesthetic technique at all times during the study.

Upon arrival in the operating room, standard monitoring equipment was applied and the systolic blood pressure(SBP), diastolic blood pressure(DBP), mean arterial pressure (MAP), heart rate (HR) and pulse oxygen saturation (SpO_2_) were recorded during the operation. The inspired oxygen and end-tidal (ET) concentrations of carbon dioxide (CO_2_) and sevoflurane were measured continuously with a calibrated (+/- 0.02% accuracy) infrared gas analyzer (Ultima, Datex, Helsinki, Finland). In addition, we used the three A-Line Auditory evoked potential index (AEPI, Danmeter, Odense, Denmark) for monitoring the anesthesia level.

Ringer's lactate solution(10 ml/kg preoperatively) was infused through a peripheral intravenous catheter. All patients were pre-oxygenated for 3 L/min via a face mask for 3 minutes prior to induction. In group T, the patients' age, height, weight and gender were entered into the TCI pump (Fresenius Orchestra Primea, Fresenius Kabi AG, Bad Homburg, Germany),and the infusion line was attached to the intravenous cannula.

Anesthesia was induced and maintained with the effect-site model of TCI with propofol and remifentanil. Propofol was monitored with the Schnider pharmacokinetic model and the remifentanil with the Minto model. The target concentrations of propofol and remifentanil were set at 3 µg/ml and 6 ng/ml during induction and were maintained at 2-3 µg/ml and 2-6 ng/ml, respectively, according to hemodynamic changes. After loss of the eyelash reflex, a bolus dose of vecuronium 0.1 mg/kg was administered; the trachea was intubated after 3 minutes.

In group S, the patients were instructed in the vital capacity breath(VCB) technique. The fresh gas flow (FGF) of the anesthesia machine was adjusted to 5 L/min oxygen, and the sevoflurane vaporizer was advanced up to 8% to provide maximum sevoflurane delivery. The reservoir bag was evacuated and allowed to refill. The face mask was placed over the nose and mouth after a forced exhalation (to residual volume), and the patients took a VCB as previously instructed. At loss of the eyelash reflex, a bolus dose of sufentanyl 0.3 µg/kg and vecuronium 0.1 mg/kg were administered, and FGF was adjusted to 2 L/min. Manually assisted ventilation was maintained with ET-sevoflurane 2% for 3 minutes, after which the trachea was intubated. The vaporizer was adjusted to sustain the sevoflurane end-tidal concentration at 1.3-2.2%according to hemodynamic changes.

MAP and HR were maintained within predetermined limits: propofol, remifentanil, ET-sevoflurane doses were adjusted to maintain the mean blood pressure within a range of ±20% of the pre-anesthesia level with HR of <100 beats/min. If relative hypertension (MAP above 20% of baseline value for > 1 minute) or tachycardia (HR>100 beats/min for >1 minute) persisted despite achievement of the maximal allowed anesthetic concentration, it was treated with perdipine 0.2 mg IV or esmolol 5 mg IV. Anesthetics were decreased only in response to a reduction of MAP to 20% of pre-induction values that was not responsive to replacement of intraoperative fluid losses (Ringer's Lactate solution 250 ml in 5-10 min). If relative hypotension (MAP below 20% of baseline value for >1 minutes) and bradycardia(HR<50 beats/min for >1 minute) persisted despite achievement of the minimal allowed anesthetic concentration, they were treated with ephedrine 6 mg IV or atropine 0.3 mg.

The sevoflurane, propofol, and remifentanil infusions continued until the last skin suture was placed. Residual neuromuscular block was pharmacologically antagonized at the end of surgery in all of the patients using neostigmine 1 mg and atropine 0.5 mg when two muscle twitches were elicited using the Neuromuscular stimulator (Hua Xiang Technology Company, HeiLongJiang Province, China). Patients were asked repeatedly in a normal tone of voice to open their eyes until an appropriate response was obtained. The trachea was extubated when a regular spontaneous breathing pattern had been re-established. After surgery, all patients were transferred to a post-anesthesia care unit (PACU). Postoperative recovery was evaluated by an independent PACU nurse blinded to the patient's allocation. In PACU, patients were also assessed for their level of pain every 30 minutes using the Visual Analog Scale (VAS). If the score was>5, we administered fentanyl 30 µg and recorded the total dosage for each patient.

The required sample size was derived from a power analysis based on the time to loss of eyelash reflex. A total of 60 patients in each group was required for a two-tailed type I error of 0.05 and a power of 90%. Data were analyzed using the SPSS statistical package, version 18.0(SPSS Inc., Chicago, IL, USA); data are expressed as means±SD, continuous variables were compared by a two-tailed student* t *test. A *x*^2 ^test or Fisher`s exact test was used to compare the two groups for qualitative variables, and p<0.05 was considered to be statistically significant.

## RESULTS

**Fig.1:**
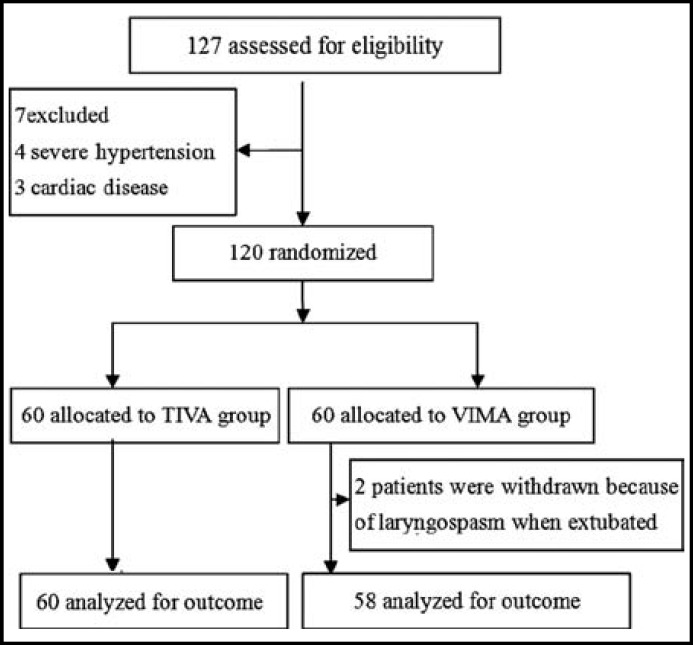
Flow diagram summarizing exclusion, allocation, withdrawal and analysis

There were no significant differences between the two groups with respect to patient characteristics, mean duration of surgery and anesthetic time (Table-I). Two patients in group S were withdrawn from the trial because of laryngospasm during tracheal extubation (Fig.1).

Time to loss of eyelash reflex was longer in group T than in group S (109±82s vs.44±11 s, p<0.001). Time to tracheal extubation was comparable between the two groups (462±129s vs.439±121 s, p=0.317). Time to eye opening and PACU duration were shorter in group T than in group S (419±134s vs.483±117s, p=0.006 and 44±15 min vs.53±24 min, p=0.017, respectively).

The incidence of pain requiring rescue medication in PACU was 53 of 60 (88%) patients in group T and 42 of 58 (70%) patients in group S (p=0.013), and the dosage of fentanyl required was higher in group T than in group S (Table-II). The incidence of Postoperation nausea and vomiting (PONV) was more frequent in group S compared with group T (20% vs. 38%, P=0.027).

During the pre- and post- incision periods, the use of ephedrine and atropine was comparable in the two groups. However, 10(17.2%) patients in group S were administered an antihypertensive drug when gallbladder issues were present, while only 1(1.7%) patient needed this drug in group T (p=0.004).

## DISCUSSION

This study demonstrates that anesthesia with TCI propofol and remifentanil or sevoflurane-sufentanyl was satisfactory when used in short-duration surgery. Induction with both propofol-remifentanil TCI and sevoflurane-sufentanyl was well tolerated. However, sevoflurane led to faster induction than TCI, which maybe a benefit for patients who need quick airway control. Previous articles reported that the induction time of sevoflurane was 90"^4^; these different outcomes may be due to the use of different methods. The speed of induction of sevoflurane is determined by whether the circuit is primed with sevoflurane, the background flow that is chosen, the effective potency of the anesthetic (what concentration must be delivered from a vaporizer to provide a clinically appropriate level of anesthesia) and the method of induction (vital capacity breath technique or tidal volume breathing).^5^^,^^6^

**Table-I:** Baseline data of two groups

	***Group T*** ***(n=60)***	***Group S*** ***(n=58)***
Age(yr) Gender(female) Weight(kg) Height(cm) Baseline BP (mmHg) Baseline of HR(beats/min) AEPI Baseline Tracheal intubation Tracheal extubation Duration of anesthesia(min) Duration of surgery(min)	44±11 68% 59±10 162±8 81±8 73±10 63±18 33±19 64±19 58±14 44±13	40.6±12 73% 59±10 161±8 83±11 75±11 65±16 33±18 60±22 56±16 43±15

Values are presented as means±SD or n(%),

No significant differences were found among the two groups

T= target-controlled infusion; S= sevoflurane

**Table-II:** Fentanyl Requirement in PACU

	***Group T*** ***(n=60)***	***Group S*** ***(n=58)***	***P value***
Use of fentanyl 30μg 60μg 90μg	53 (88%) 12 (23%) 22 (42%) 19 (35%)	42 (70%) 23 (55%) 15 (35%) 4 (10%)	0.013 0.027 0.166 0.001

Value are presented as n(%). T= target-controlled infusion; S= sevoflurane.

In terms of recovery profile, faster awakening in the propofol–remifentanil group most likely was caused by a more rapid elimination of remifentanil rather than propofol, compared with that of sevoflurane. The time of stay in PACU was longer in group S mainly because of a higher incidence of PONV, which required more time to address.

Pain is an important patient complaint in the postoperative period, and the dosage of analgesia was significantly higher in group T when patients stayed in PACU. This outcome may have occurred for two reasons：the first is that, in group T, we used remifentanil for analgesia, which is a short-acting opioid and the context-sensitive half-time is constant regardless of the duration of infusion; the second reason may be due to high doses, long-term treatment, or abrupt changes in the concentrations of the opioid, especially, remifentanil, which may have led to hyperalgesia.^7^ Therefore, we were reminded that an effective analgesic protocol needs to be established during the perioperative period, especially when using remifentanil.

PONV has been another major morbidity for ambulatory surgery. In fact, vomiting is the most important factor from a patient’s point of view. PONV was more common in group S. The experienced anesthesiologists believe that the incidence of PONV is related to the amount of postoperative opioids that are administered.^8^ Although group T required more fentanyl in PACU for pain control, the incidence of PONV was still lower in group T. One explanation may be that the patients in group S did not receive propofol, which has antiemetic properties.^9^ Another explanation may be that sevoflurane causes a greater incidence of PONV.^10^ The PONV may be a function of the initial high concentration of sevoflurane or it may have been caused by air and gases, which may be swallowed into the stomach during induction. Therefore, it may be prudent to give prophylactic antiemetics to patients receiving inhaled induction with sevoflurane.

Differences were observed in hemodynamic changes between the two groups. We recorded that 10 (17.2%) patients had experienced hypertensive episodes when gallbladder issues were present in group S. The current study demonstrates that the AEPI can be a guide to the depth of sedation and movement in response to skin incision during sevoflurane anesthesia.^11^ Theoretically, for meeting the surgical needs, the AEPI should below 30. Although the AEPI of these 10 patients ranged from 13-93, six of them exceeded 30. However, the AEPI sometimes exhibited artifact signals and poor anti-interference ability when using the electrical surgery unit, but we did not observe awareness in these six patients during surgery. Therefore, we should combine the other index to judge the level of anesthesia.^12^ At that moment, ET-sevoflurane of these ten patients ranged from 2.0-2.2. Moreover, we used 0.3 µg/kg sufentanyl; therefore, the anesthesia level was beyond AD_95 _(1.3 MAC). At the beginning of the surgery, 0.3 µg/kg sufentanyl can effectively inhibit noxious stimulation due to tracheal intubation, incision, and CO_2_ inflation. However, together with the metabolism of the sufentanyl, during the late phases of surgery, we mainly rely on the sevoflurane to maintain the anesthesia. If the sympathetic responses are totally depressed, the anesthesia level should have attained MAC_bar _(MAC_bar_ is defined as the point when the inhaled anesthetics at a given minimum alveolar anesthetic concentration block the adrenergic response to a surgical incision in 50% of patients), and average concentration of ET-sevoflurane should achieve approximately 4.1%.^13^ However, we did not administer higher concentrations of sevoflurane, which could suppress blood pressure and heart rate responses in all patients and lead to the risk of excessive hypotension. The clinical implication of these findings was that the induction combined with 0.3 µg/kg sufentanyl was sufficient for analgesia and inhibiting the sympathetic response; however, when the plasma concentration was reduced in the mid/late phases of surgery, it may require another mini-dose of sufentanyl for meeting the requirements of surgery. Of course, this consideration requires further research.

This study had some limitations. First, the study was randomized but it was difficult to blind the personnel who made intraoperative observations; therefore, there was a possibility of observer bias. Second, if we had implemented preemptive analgesia, it would have more consistent with the demands of ethics. Finally, the study lacked a reliable depth of anesthesia monitors, as both AEPI and BIS has shortcomings.^14^

## CONCLUSION

The main findings of this study is that none of the techniques used was superior, with each having advantages and disadvantages for patients undergoing laparoscopic cholecystectomy. Thus, for each patient and each procedure, the anesthesiologist must weigh the risks, benefits and the patient’s will to choose the appropriate anesthetic technique.

## Notes

***Source of funding:*** This work was supported in part by National Natural Science Foundation of China (NSFC 81200865).
